# Molecular Consequences of the Myopathy-Related D286G Mutation on Actin Function

**DOI:** 10.3389/fphys.2018.01756

**Published:** 2018-12-04

**Authors:** Jun Fan, Chun Chan, Elyshia L. McNamara, Kristen J. Nowak, Hiroyuki Iwamoto, Julien Ochala

**Affiliations:** ^1^Department of Physics and Materials Science, The University of Hong Kong, Hong Kong, Hong Kong; ^2^City University of Hong Kong Shenzhen Research Institute, Shenzhen, China; ^3^Harry Perkins Institute of Medical Research, QEII Medical Centre, The University of Western Australia, Nedlands, WA, Australia; ^4^Department of Health, Office of Population Health Genomics, Public and Aboriginal Health Division, Government of Western Australia, East Perth, WA, Australia; ^5^School of Biomedical Sciences, Faculty of Health and Medical Sciences, The University of Western Australia, Crawley, WA, Australia; ^6^SPring-8, Japan Synchrotron Radiation Research Institute, Sayo, Japan; ^7^School of Basic and Medical Biosciences, Faculty of Life Sciences and Medicine, King’s College London, London, United Kingdom

**Keywords:** actin mutation, myopathy, muscle force, actin mechanics, muscle fiber

## Abstract

Myopathies are notably associated with mutations in genes encoding proteins known to be essential for the force production of skeletal muscle fibers, such as skeletal alpha-actin. The exact molecular mechanisms by which these specific defects induce myopathic phenotypes remain unclear. Hence, in the present study, to better understand actin dysfunction, we conducted a molecular dynamic simulation together with *ex vivo* experiments of the specific muscle disease-causing actin mutation, D286G located in the actin-actin interface. Our computational study showed that D286G impairs the flexural rigidity of actin filaments. However, upon activation, D286G did not have any direct consequences on actin filament extension. Hence, D286G may alter the structure of actin filaments but, when expressed together with normal actin molecules, it may only have minor effects on the *ex vivo* mechanics of actin filaments upon skeletal muscle fiber contraction.

## Introduction

Congenital myopathies are genetic muscle diseases that start at a very young age. Patients experience weakness in the limb, masticatory and respiratory muscles which can result in severe debilitation, loss of independence and the need for constant care (Nowak et al., 2013; Tajsharghi and Oldfors, 2013). The related medical expenses and economic consequences are therefore enormous (Nowak et al., 2013; Tajsharghi and Oldfors, 2013). Hence, it is of vital importance to get a detailed understanding of the molecular pathophysiology for this class of disorders.

Over the last 20 years, a large number of reports have associated these congenital myopathies with mutations in genes encoding proteins fundamental for myofiber structure and function (Nowak et al., 2013; Tajsharghi and Oldfors, 2013). Among these genes is *ACTA1*, which encodes the skeletal muscle protein alpha-actin (Nowak et al., 2013). Actin is essential for muscle contraction and force generation. Actin monomers of the thin filaments directly interact with myosin molecules to form cross-bridges that initiate force development and motion at the sarcomere and myofiber levels (Gordon et al., 2000). Missense *ACTA1* mutations, and thus, single amino acid replacements in actin proteins, are now known to depress myosin cross-bridge formation and myofiber force production explaining in part why patients experience muscle weakness and have myopathic phenotypes (Lindqvist et al., 2012, 2013, 2016; Ochala et al., 2012, 2015).

When carefully looking at the 200 different single residue substitutions in actin inducing inherited myopathies, only a small portion of these is located on amino acids directly interacting with myosin, or with tropomyosin and/or troponin molecules, which are also involved with actin binding. It is then unclear how the remaining defects interfere with cross-bridge functioning (Nowak et al., 2013). In the present study, based on one of our previous studies (Chan et al., 2016) and on computational simulations (Kojima et al., 1994; Wakabayashi et al., 1994; Miller et al., 2010) we aimed to test the hypothesis that one of the major triggering factors of muscle weakness would be altered actin filament structure. To verify this, we applied a unique combination of molecular dynamics (MD) simulation and *ex vivo* experiments (small-angle x-ray scattering). We focused our attention on D286G which is located in subdomain 3 at the actin-actin interface (Ravenscroft et al., 2011a). Even though D286G does not have any known interaction with myosin, tropomyosin and/or troponin molecules, it has been linked to improper myosin binding (Ochala et al., 2012, 2015) and nemaline myopathy in mice and humans (Ravenscroft et al., 2011a).

## Materials and Methods

### Animals

After sacrifice, skeletal muscles (extensor digitorum longus, EDL) were dissected from 3 to 4-month old male wild-type and age-matched transgenic mice expressing the *ACTA1* D286G mutation. For a complete description of the transgenic mice, please refer to Ravenscroft et al. (2011a). All procedures involving animal care, welfare and handling were performed according to institutional guidelines. They were reviewed and approved by the Animal Ethics Committee of The University of Western Australia (RA03/100/918).

### Muscle Preparation and Fiber Permeabilization

EDL muscles were placed in relaxing solution at 4°C. Bundles of approximately 50 myofibers were dissected free and then tied with surgical silk to glass capillary tubes at slightly stretched lengths. They were then treated with membrane-permeabilizing solution (relaxing solution containing glycerol; 50:50 v/v) for 24 h at 4°C, after which they were transferred to -20°C. In addition, the muscle bundles were treated with sucrose, a cryo-protectant, within 1–2 weeks for long-term storage (Frontera and Larsson, 1997). They were detached from the capillary tubes and snap frozen in liquid nitrogen-chilled isopentane and stored at -80°C.

### Small-Angle X-Ray Scattering and Analyses

Two to three days prior to X-ray recordings, bundles were de-sucrosed, transferred to a relaxing solution and single myofibers were dissected. Arrays of approximately 30 myofibers were set up. For each myofiber, both ends were clamped to half-split gold meshes for electron microscopy (width = 3 mm), which had been glued to precision-machined ceramic chips (width = 3 mm) designed to fit to a specimen chamber. The arrays were then transferred to the skinning solution and stored at -20°C. Approximately 80 arrays were mounted (10 arrays per mouse – four knock-in and four wild-type mice – corresponding to approximately 2,400 attached fibers) (Ochala et al., 2015; Chan et al., 2016).

On the day of X-ray recordings, arrays were placed in a plastic dish containing a pre-activating solution and washed thoroughly to remove the glycerol. They were then transferred to the specimen chamber, capable of manual length adjustment and force measurement (force transducer, AE801, Memscap, Bernin, France), filled with a pre-activating solution. Mean sarcomere length was measured (laser diffraction) and set to 2.50 μm. Subsequently, X-ray diffraction patterns were recorded at 15°C, first in the pre-activating solution and then in the activating solution (pCa 4.5) when maximal steady-state isometric force was reached.

For each array, approximately 20 to 30 diffraction patterns were recorded (depending on myofiber length) for each solution at the BL45XU beamline of SPring-8. The wavelength was 0.1 nm, and the specimen-to-detector distance was 3.47 m to maximize the spatial resolution to determine filament extension. As a detector, a cooled CCD camera (C4880, Hamamatsu Photonics; 1000 × 1018 pixels) was used in combination with an image intensifier (VP5445, Hamamatsu Photonics). To minimize radiation damage, the exposure time was kept low (1–2 s) and the specimen chamber was moved by 100 μm after each exposure. Moreover, we placed an aluminum plate (thickness, 0.35–0.5 mm) upstream of the specimen chamber. Following the x-ray recordings, background scattering was subtracted, and reflection intensities were determined as described elsewhere. Note that in the literature, the determination of actin spacing (or actin extensibility) is historically performed using actin layer line (ALL) reflections, that is, the 6th ALL (ALL6, *d* = 5.9 nm) and 7th ALL (ALL7, *d* = 5.1 nm) (Wakabayashi et al., 1994). However, using ALL6 and ALL7 to assess the actin spacing leads to errors (Chan et al., 2016). Hence, here, we used the 3rd order meridional reflection of troponin (TN3, *d* = 12.9 nm) which has a stable intensity in membrane-permeabilized myofibers set at a define sarcomere length (Chan et al., 2016).

Relaxing and activating solutions contained 4 mM Mg-ATP, 1 mM free Mg^2+^, 20 mM imidazole, 7 mM EGTA, 14.5 mM creatine phosphate, 324 U/mL creatine phosphokinase, 1000 U/mL catalase, and KCl to adjust the ionic strength to 180 mM and pH to 7.0. Dithiothreitol (DTT) was also added (10 mM). The pre-activating solution was identical to the relaxing solution, except that the EGTA concentration was reduced to 0.5 mM. The concentrations of free Ca^2+^ were 10^-9.0^ M (relaxing and pre-activating solutions, pCa 9.0) and 10^-4.5^ M (activating solution, pCa 4.5).

### Molecular Dynamics Simulation

One actin filament containing the D286G mutants composed of subunits with bound Mg^2+^-ADP was constructed. This was based on vertebrate (rabbit) skeletal muscle actin Protein Data Bank entry 2ZWH, with a high-affinity Mg^2+^ cation placed at the nucleotide-binding site and the first solvation shell of explicit waters included. The filament contained 13-monomer subunits as described in Saunders and Voth (2011); Fan et al. (2012, 2013). The filament was solvated in explicit TIP3P water molecules, with K^+^ and Cl^-^ ions included at a final concentration of 0.18 M using the solvate and autoionize tools in VMD (Humphrey et al., 1996). Periodic boundary conditions were introduced such that the filament was aligned to repeat along the z direction, interacting with itself at the box edges.

The MD simulations were performed using NAMD 2.10 package (Phillips et al., 2005) and the CHARMM 22/27 force field (MacKerell et al., 1998) with CMAP correction (Mackerell et al., 2004). Electrostatic interactions were calculated using the particle mesh Ewald sum method with a cutoff of 12 Å. All hydrogen-containing covalent bonds were constrained by the SHAKE algorithm, therefore allowing an integration time step of 2 fs. Before production runs, an actin filament system was energy minimized, heated, and pre-equilibrated for 100 ps in the canonical ensemble while the protein backbone, the nucleotide, the active site Mg^2+^, and water oxygen atoms were harmonically restrained with spring constant 1 kcal/mol Å^2^. Simulations were then continued in the constant NPT ensemble (310 K and 1 atm) for an additional 200 ps. Langevin thermostats with a damping coefficient of 0.5 ps^-1^ were used to control the system temperature. A Langevin-piston barostat with a piston period of 2 ps and a damping time of 2 ps was used to control the pressure. Constraints were next released step-wise (with spring constant gradually decreased from 1 kcal/mol Å^2^ to 0 by steps of 0.1 kcal/mol Å^2^) over a total of 100 ps before starting the production runs. A total of 166 ns of data were generated for the D286G actin filament systems. Only data after the system was equilibrated (after 75 ns production run) were taken for further analysis. The final 90 ns of data were used for analysis unless otherwise specified. All quantities presented in this article are the averaged value over all subunits and the simulation windows. All MD data are analyzed based on the block averages method (Frenkel and Smit, 2002). All errors in the MD results section refer to standard deviation (SD).

### Statistics

The unpaired Student’s *t*-test was applied, and in cases where the data did not meet the criteria of normality (Kolmogorov–Smirnov test, *p* < 0.05), the non-parametric Mann-Whitney rank-sum test was performed.

## Results and Discussion

In the present work, we studied a missense *ACTA1* mutation resulting in the substitution of one single amino acid in the skeletal alpha-actin protein (D286G, according to the standard Human Genome Variation Society nomenclature) (Ravenscroft et al., 2011a). By using a MD simulation, together with *ex vivo* experiments consisting of X-ray diffraction, we observed that D286G alters actin filamentous structure. We also showed that, in contracting skeletal muscle fibers expressing both mutant and normal actin monomers, such structural defects do not have any major toxic consequences on actin mechanics.

### Actin Filaments Have Altered Flexural Rigidity in the Presence of D286G

Even though we did not observe any change in the internal structure of individual actin monomers with D286G, interestingly, we found that intra-filament distances between monomers were affected by the residue replacement. Indeed, most of the longitudinal and lateral contact distances were lower in filaments carrying D286G than in WT filaments (Table 1). This is in agreement with another amino acid substitution in the actin protein (H40Y) leading to myopathic features in mice and humans (Lindqvist et al., 2013; Nguyen et al., 2011). In the presence of D286G or H40Y, the DNase I-binding loop (or D-loop, residues 38–52) appears to be involved in many of the alterations. This particular loop plays a key role in the actin-actin interface as it directly interacts with the C-terminus of the adjacent subunit in actin filament (Dominguez and Holmes, 2011). Taken together, our data indicate that in the presence of D286G or H40Y, individual actin monomers become closer.

**Table 1:** Comparison of longitudinal and lateral contacts between filaments containing D286G and wild-type (WT) filaments.

		Equilibrated distance (A)	
	Residue ID	D286G filament	WT filament^a^
***Longitudinal contact***			
D-loop to C-term	41 vs. 374	12.51 (1.40)	13.09 (1.50)
	61 vs. 374	22.78 (0.82)	23.29 (1.24)
	45 vs. 370	14.68 (1.54)	14.85 (2.09)
D-loop to SD1	45 vs. 169	8.36 (1.73)	9.74 (1.74)
	61 vs. 169	12.85 (0.81)	12.78 (1.40)
D-loop to SD3	62 vs. 288	7.52 (0.77)	7.52 (1.13)
SD4 to SD3	205 vs. 286	9.27 (0.73)	9.29 (0.53)
	241 vs. 322	11.41 (1.74)	11.03 (1.76)
***Lateral contact***			
H-plug vs. C	265 vs. 374	19.35 (0.98)	20.13 (1.15)


*Standard deviations are included between brackets. C-term, C-terminus; D-loop, DNase I-binding loop; SD1, subdomain 1; SD3, subdomain 3; and SD4, subdomain 4. ^a^Data from Fan et al. (2013).*

To further investigate intra-filament changes, we assessed the subunit geometries through coarse-graining residues into relatively rigid sub-groups: residues 5–33, 80–147, and 334–349 as SG1; residues 34–39 and 52–69 as SG2; residues 148–179 and 273–333 as SG3; residues 180–219 and 252–262 as SG4. For the analysis, we referred the centres of geometry (COGs) of these residues as R1, R2, R3, and R4 (Chan et al., 2016; Table 2). We then noticed a few differences between filament subunits carrying D286G and WT filament subunits. The equilibrated R2–R1–R3 angles as well as R2–R1–R3–R4 dihedral angles were greater in filament subunits containing D286G than in WT filament subunits. This is in line with differences reported with H40Y (Chan et al., 2016). All these findings indicate a change in the actin filament stability in the presence of D286G or H40Y that is related to modifications in the interactions between the D-loop and adjacent subunits.

**Table 2:** Coarse-grained (CG) representation of subunit geometries in filaments expressing D286G and wild-type (WT) filaments.

	Parameters	D286G equilibrated	WT equilibrated^b^
***Bond (A)***	R1–R2^a^	21.84 (0.39)	22.99 (0.39)
	R1–R3	24.92 (0.18)	24.85 (0.28)
	R3–R4	24.87 (0.19)	24.97 (0.28)
***Angle (°)***	R1–R3–R4	74.74 (1.83)	74.42 (1.96)
	R2–R1–R3	105.95 (1.57)	102.06 (2.06)
***Dihedral (°)***	R2–R1–R3–R4	12.01 (3.36)	10.27 (3.41)


*Standard deviations are included between brackets. ^a^Residue sets: R1: 5–33, 80–147, and 334–349; R2: 34–39 and 52–69; R3: 148–179 and 273–333; R4: 180–219 and 252–262. ^b^Data from Fan et al. (2013).*

Finally, we calculated actin filament persistence length using MD simulation (Supplementary Figure 1). This parameter was larger (*p* < 0.002) in filaments containing D286G than in wild-type (WT) filaments (17.2 ± 6.0 and 9.80 ± 0.14 μm, respectively). The increase in the persistence length of actin filament demonstrates that D286G stiffens the filament. In other words, the filament resilience increases with this mutation. To understand the potential mechanisms, we measured the crossover length of the filament with thirteen D286G subunits. The crossover length was 356.85 ± 0.71 Å, which was smaller than that of WT filament (365 ± 15 Å) with *p* < 0.00007 (McGough et al., 1997). Note that as our calculations did not include phalloidin or other actin-binding proteins known to stabilize the filaments, our WT value was shorter than the ones previously reported (Yanagida et al., 1984; Gittes et al., 1993; Ott et al., 1993).

We conclude that based on our data analysis, D286G results in a decrease in the distance between the D-loop and the C-terminal region as well as in the crossover length. It also induced an increase in the persistence length. As this persistence length spans beyond the distance between adjacent subunits, it is likely that a cooperative mechanism between subunits exists in the presence of D286G allowing a negative propagation to distant subunits.

### Thin Filament Extension Upon Activation Is Not Altered When D286G Is Expressed Together With Normal Actin Monomers

As the above results from MD simulations imply that actin filaments may have affected stabilities we measured their maximal extensibility by recording and analyzing the actin spacing via X-ray diffraction patterns. Relaxed and activated single membrane-permeabilized myofibers from WT mice and mice expressing both D286G and WT actin molecules were tested. These experiments were possible, as muscles from transgenic mice do not have any signs of sarcomeric ultrastructure (Ravenscroft et al., 2011b).

We determined the actin spacing by using the third order meridional reflection of troponin (TN3, *d* = 12.9 nm). As previously reported (Chan et al., 2016) we subtracted the background scattering, and then fitted the intensity profile of TN3 to the Gaussian function (with its center being the position of the peak). Data in the ranges of ±4 pixels as well as ±3 pixels from the observed peak were used (Chan et al., 2016). To gain a reasonably high signal-to-noise ratio and to make the curve fitting reliable, in the present study, we used a qualitative approach where all the data obtained for all the arrays for each group of mice were sum up. We then compared the mean values. Hence, upon maximal activation, in WT myofibers, the peak shifted by ∼0.34 and ∼0.31%, respectively (Supplementary Figure 2). Interestingly, in myofibers carrying D286G, the peak only moved by ∼0.18 and ∼0.13%, respectively (Supplementary Figure 2). Hence, in contracting muscle fibers expressing D286G, the actin filament extension appeared reduced by ∼50%. Actin filament extensibility depends on myosin binding as well as on force production (Gordon et al., 2000). As in the presence of D286G, muscle fiber force generation is reduced by ∼50% (Ochala et al., 2012) it is unlikely that our experimental differences arise from a direct effect of the mutation on the ability of actin filament to change their conformation during activation. This result differs from the findings related to the H40Y amino acid substitution (Chan et al., 2016). Several potential alternatives may underlie this. First, it is plausible that the changes in actin filament structure in the presence of D286G are less damaging than H40Y. Second, as the amount of mutant actin molecules in the myofibers used for our current experiments is ∼25% (Ochala et al., 2012) and as normal and D286G actin molecules are likely to form heteropolymers, significant differences may be hindered.

## Conclusion

To conclude, our computational study reveal that D286G disrupts the D-loop interaction with neighboring actin monomers, affecting the flexural rigidity. Despite this, when D286G is mixed with its wild-type form, our *ex vivo* experiments indicate that the mechanics of actin filaments is not directly altered.

## Author Contributions

JF, KN, HI, and JO contributed to the conception and design of the work. JF, CC, EM, KN, HI, and JO did the acquisition, analysis, and interpretation of data, drafted the work and revised it critically, approved the final version to be published, and agreed on all aspects of the work.

## Conflict of Interest Statement

The authors declare that the research was conducted in the absence of any commercial or financial relationships that could be construed as a potential conflict of interest.
