# Supplementary material for: Molecular Consequences of the Myopathy-Related D286G Mutation on Actin Function
Source: Front Physiol. 2018 Dec 4;9:1756. doi: 10.3389/fphys.2018.01756 (PMC6288369; doi:10.3389/fphys.2018.01756)

**Supplementary material: Molecular consequences of the myopathy-related D286G mutation on actin function**

**Supplementary Figure 1.** Molecular dynamics simulation setup

Actin subunit structure with rigid portions of SG1, SG2, SG3, and SG4 shown in pink, blue, purple, and red, respectively, and the COG of each rigid group is represented in a sphere of the same colour, labelled as R1, R2, R3, and R4. (A) Upper panel is the subunit crystal structure (PDB ID: 2ZWH) and the lower panel is the equilibrated D286G structure after molecular dynamics simulation. Subunit structure D-loop is shown and labelled in yellow. (B) represents an effective infinitely long D286G actin filament in a periodic boundary condition (PBC) water box under physiological conditions.


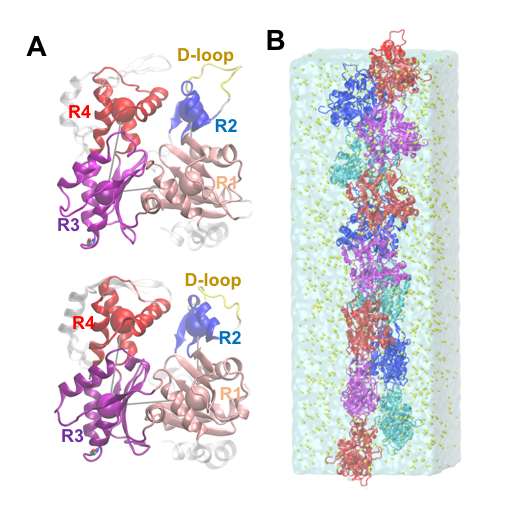


**Supplementary Figure 2.** TN3 intensity profiles

X-ray diffraction patterns (specimen-to-detector distance: 3.47 m) of membrane-permeabilised myofibers set at a sarcomere length of 2.50 µm from D286G mice in resting (pCa 9.0, blue lines on graphs) and maximal activating (pCa 4.5, red lines on graphs) conditions. The thinner red and blue curves represent the observed data, and the thicker red and blue curves represent the fitted Gaussian function with a range of fitting ± 4 pixels or ± 3 pixels.


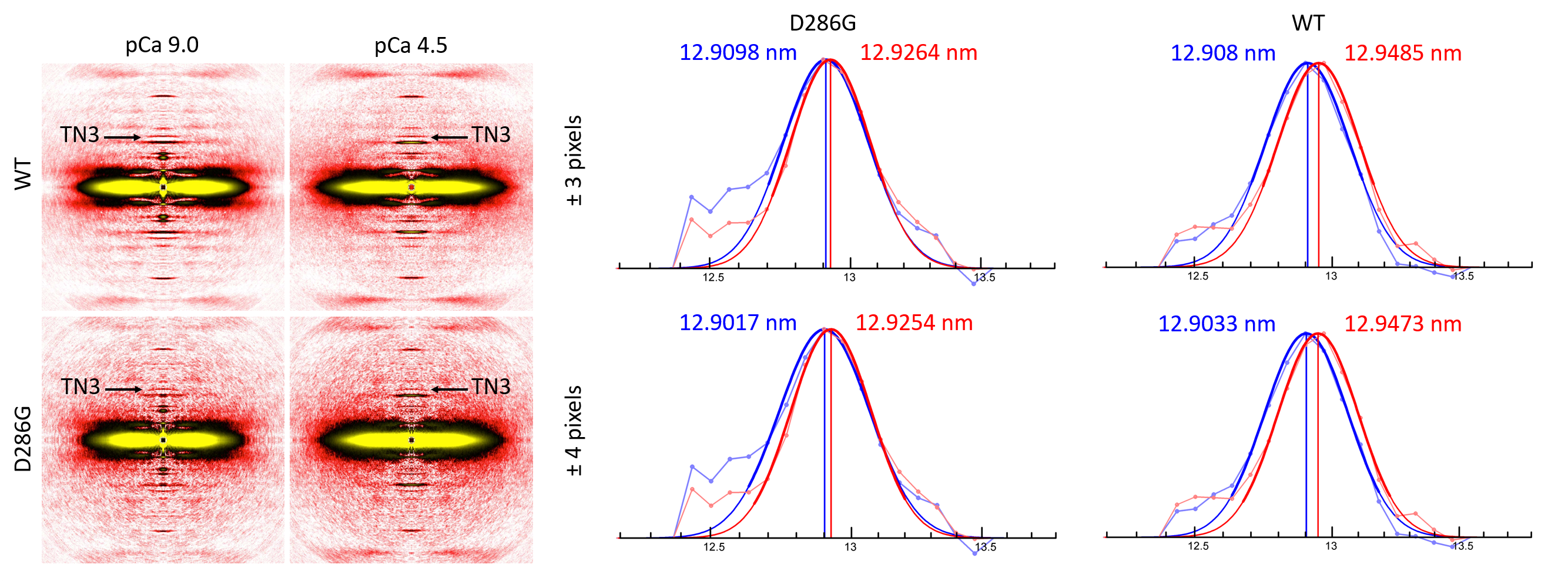

Supplement: Supplementary file 1 [file Data_Sheet_1.doc]
